# What Do We Mean When We Say “AI?” Why Context—Not Volume—Should Guide Emergency Medicine

**DOI:** 10.1016/j.acepjo.2025.100309

**Published:** 2025-12-26

**Authors:** Christian Rose, Carl Preiksaitis

**Affiliations:** Department of Emergency Medicine, Stanford University School of Medicine, Stanford, California, USA

## 1 Introduction

Artificial intelligence (AI) is no longer a speculative disruptor; it is woven into daily emergency care. However, as AI spreads, a basic question remains: what exactly counts as “AI,” and in what context is it being used? For some clinicians, “AI” refers to large-language-model assistants, and for others, to regression-based calculators, imaging classifiers, or predictive dashboards. Treating those heterogeneous tools as a single category obscures the real question for emergency medicine (EM): which tool, for which task, on which data, at what stakes, and with what oversight? Until we define AI by the context of use, adoption statistics, disclosure requirements, and governance will talk past practice and risk misdirecting improvement efforts.

Shy’s convenience survey of American College of Emergency Physicians (*ACEP*) members offers a useful snapshot.^1^ Respondents reported noninstitutional AI use in roughly one-third of cases and institutional AI for just over half across multiple applications. Attitudes were mixed but generally optimistic: most anticipated efficiency gains and many expected better care quality; however, these beliefs preceded systematic outcome measurement, and fewer than half voiced concern about bias, Health Insurance Portability and Accountability Act compliance, or job displacement.^1^

## 2 A Definition Problem

Those findings reveal a fundamental measurement problem. Without a shared operational definition of “AI” and a way to mark context, “adoption” rates inevitably blend perception with practice. A clinician may not call PECARN or NEXUS “AI,” although both derive from supervised learning, but the same clinician may count asking a large language model to draft discharge notes as “AI use.” The survey checkbox thus captures behaviors with very different risk profiles, oversight needs, and workflow implications. This ambiguity reveals what we must begin to capture: the context of use, not just adoption rates.

Current policies make addressing this ambiguity more pressing. Pennsylvania’s HB 1925, for example, would require patient disclosure when facilities use “machine-based” systems to make predictions, recommendations, or decisions.^2^ Read plainly, the bill’s definition is expansive enough to sweep in long-standing statistical calculators alongside modern predictive and generative systems. That would numb patients with boilerplate notices while obscuring the higher-stakes deployments that warrant informed discussion—a form of compliance theater that satisfies regulators without serving patients. A more clinically meaningful standard is context-triggered disclosure keyed to stakes: direct impact on clinical decision-making, the handling of protected health information outside the electronic health record boundary, and the degree of automation rather than a label triggered merely by the word “AI.”^2^

## 3 Same Tool, Different Stakes

Context determines risk in ways that volume-based thinking cannot capture. The same model can be appropriate in one scenario and problematic in another. Imagine using a language model to summarize triage complaints and help a nurse prioritize rooms. If used as an aid with clear human oversight, it is ethically distinct from the same model used to summarize a visit and autogenerate discharge instructions, although both may be at risk of propagating an unverified diagnosis. The model did not change; the use-case did. Contemporary risk frameworks converge on this point: the National Institute of Standards and Technology’s AI Risk Management Framework and the EU AI Act both evaluate systems by intended purpose and context, calibrating controls accordingly.^3^^,^^4^

If EM reported and studied AI in context this way our evidence would travel farther and allow meaningful comparison between deployments. Shy’s study survey surfaces a tension familiar to emergency physicians: the promise of efficiency gains competing with concern about algorithmic bias, but framing these as competing values creates a false choice. Context-based measurement resolves this by treating efficiency and safety as paired outcomes that must be evaluated together. Claims of faster documentation, smoother triage, or shorter length of stay should be reported alongside sentinels for safety and equity, such as diagnostic-error proxies, alert-override patterns, and subgroup performance. Governance details like who owns the model or how often it is audited belong in the same paragraph as time-savings claims. Consistently publishing these gains together with safety and equity indicators would reward disciplined integration rather than simply the volume of adoption.^1^

## 4 What Success Actually Requires

AI in EM is not a monolith. It is a suite of evolving tools entering the cognitive and operational fabric of care. Surveys must move past the binary “Do you use AI?” and instead describe what kind of tool is being used, for what purpose, at what point in the workflow, on what data, and under what supervision. Before we can measure success, we must decide what behaviors we are counting and in what context. The most useful question for the next phase is “Which tool, for which patient, in which workflow, and at what acceptable trade-off?” Until we answer explicitly, transparently, and empirically—which tool, for which patient, in which workflow, and at what acceptable trade-off—EM risks embracing tools that accelerate workflow but erode safety, and vice versa, hindering rather than advancing the very care it aims to transform.

## Funding and Support

By *JACEP Open* policy, all authors are required to disclose any and all commercial, financial, and other relationships in any way related to the subject of this article as per ICMJE conflict of interest guidelines (see www.icmje.org). The authors have stated that no such relationships exist.

## Declaration of Generative AI And AI-Assisted Technologies IN The Manuscript Preparation Process

During the preparation of this work, the author(s) used Claude (Anthropic) in order to edit and revise elements for length. After using this tool/service, the author(s) reviewed and edited the content as needed and take(s) full responsibility for the content of the published article.
